# Association between breastfeeding and preeclampsia in parous women: a case –control study

**DOI:** 10.1186/s13006-021-00391-3

**Published:** 2021-06-29

**Authors:** Ishag Adam, Duria A. Rayis, Nadiah A. ALhabardi, Abdel B. A. Ahmed, Manal E. Sharif, Mustafa I. Elbashir

**Affiliations:** 1 Department of Obstetrics and Gynecology, Unaizah College of Medicine and Medical Sciences, Qassim University, Unaizah, Saudi Arabia; 2 Faculty of Medicine, University of Khartoum, P.O Box 102, Khartoum, Sudan; 3 College of Medicine, King Khalid University, Abha, Saudi Arabia

**Keywords:** Preeclampsia, Breastfeeding, Risk factors, Parous women, Sudan

## Abstract

**Background:**

Preeclampsia is a global health problem and it is the main cause of maternal and perinatal morbidity and mortality. Breastfeeding has been reported to be associated with lower postpartum blood pressure in women with gestational hypertension. However, there is no published data on the role that breastfeeding might play in preventing preeclampsia. The aim of the current study was to investigate if breastfeeding was associated with preeclampsia in parous women.

**Method:**

A case-control study was conducted in Saad Abualila Maternity Hospital in Khartoum, Sudan, from May to December 2019. The cases (*n* = 116) were parous women with preeclampsia. Two consecutive healthy pregnant women served as controls for each case (*n* = 232). The sociodemographic, medical, and obstetric histories were gathered using a questionnaire. Breastfeeding practices and duration were assessed.

**Results:**

A total of 98 (84.5%) women with preeclampsia and 216 (93.1%) women in the control group had breastfed their previous children. The unadjusted odds ratio (OR) of preeclampsia (no breastfeeding vs breastfeeding) was 3.55, 95% confidence interval (CI) 1.64,7.70 and *p* value = 0.001 based on these numbers. After adjusting for age, parity, education level, occupation, history of preeclampsia, history of miscarriage, body mass index groups the adjusted OR was 3.19, 95% CI 1.49, 6.82 (*p* value = 0.006).

**Conclusion:**

Breastfeeding might reduce the risk for preeclampsia. Further larger studies are required.

## Background

Preeclampsia is characterized by new-onset hypertension and proteinuria after the 20th week of gestation, and it is one of the main pregnancy-related disorders [1]. It has been estimated that approximately 2–8% of pregnant/parturient women worldwide have preeclampsia [2]. Preeclampsia is a main cause of maternal and perinatal morbidity and mortality [2]. The function of multiple systems and organs, such as the liver, kidneys, and brain, can be affected by preeclampsia [2]. The exact aetiology and pathophysiology of preeclampsia are not well understood; however, several risk factors (parity, body mass index (BMI), chronic disease, lack of antenatal care, and anaemia) have been identified as risk factors and can be used as predictors of preeclampsia [3].

Breastfeeding is globally considered a vital public health issue with social, religious, and economic implications [4, 5]. Breastfeeding is associated with reduced negative cardiovascular sequelae that are attributed to preeclampsia (hypertension, type II diabetes, metabolic syndrome, and cardiovascular disease) [6]. It has recently been shown that women who had normotensive pregnancies and breastfed were at a lower risk of atherosclerosis than peers who had never breastfed [7]. It has been shown that breastfeeding is associated with lower blood pressure in later life [8]. Moreover, breastfeeding was reported to be associated with lower postpartum blood pressure in women with normal weight [9] and in women with gestational hypertension [10]. To the best of our knowledge, there is no published data on the role that breastfeeding might play in preventing preeclampsia. Hence, this study was conducted in Khartoum, Sudan, to investigate if breastfeeding was associated with preeclampsia in parous women. Preeclampsia is the leading cause of poor maternal and perinatal outcomes in Sudan [11]. We have previously showed that several clinical, biochemical, and genetic factors are associated with preeclampsia [12–16]. However, the real effects of breastfeeding in reducing the risk of preeclampsia remain debated.

## Methods

A case-control study was conducted in Saad Abualila Maternity Hospital in Khartoum, Sudan, from May to December 2019. The cases were parous women with preeclampsia recruited from the labour ward. The American College of Obstetricians and Gynaecologists criteria [1] were used to diagnose preeclampsia as follows: pregnant women with a blood pressure equal to or greater than 140/90 mmHg on two occasions at least 6 h apart plus proteinuria (≥ 300 mg/24 h). Women with a blood pressure ≥ 160/110 mmHg on two occasions, proteinuria of ≥5 g/24 h, and HELLP syndrome (haemolytic anaemia, elevated liver enzymes, and low platelet count) were considered to have severe preeclampsia, otherwise, preeclampsia was considered mild [1]. Women who presented with preeclampsia before and after 34 weeks were classified as having early-onset and late-onset preeclampsia, respectively [17]. Two consecutive healthy pregnant women without any underlying diseases, such as hypertension, renal disease, diabetes, or proteinuria, served as controls for each case. Women with multiple pregnancies or other comorbidities, such as diabetes mellitus, renal disease, thyroid disease, major foetal anomalies, foetal demise, and haemolytic disease, were excluded from the study.

After providing an informed consent, women (in the case and control groups) were asked about their age, parity, educational level, antenatal attendance, interpregnancy interval (IPI), history of miscarriage, and history of preeclampsia/hypertension in previous pregnancies. Pre-pregnancy or first-trimester weight and height were used to compute the BMI as weight/height^2^. The BMI was classified per the World Health Organization classification for BMI [18] as follows: normal weight (18.5–24.9 kg/m^2^), overweight (25.0–29.9 kg/m^2^), or obese (30.0–34.9 kg/m^2^). The haemoglobin concentration (at delivery) and blood group were recorded. Following delivery, the gender of the newborn was recorded.

To assess breastfeeding practices and duration, women were initially asked “Did you breastfeed your last baby?” Women who answered “no” were categorized as never breastfed.

The sample included 116 cases and 232 controls (ratio of 1:2). The sample size was calculated based on the report on breastfeeding (87.2% of women initiated breastfeeding within the first hour) in eastern Sudan [19]. We assumed that 85% of the cases (preeclampsia) vs 95% of the controls had breastfed their babies (regardless of the duration of breastfeeding). This sample size would result in 80% power with a precision of 5% to detect a difference of 10% in breastfeeding rates (95% vs 85%) between mothers with and without preeclampsia.

### Statistical analysis

Statistical analysis was performed using Statistical Package for the Social Sciences (SPSS) for Windows version 22.0 (SPSS Inc., Chicago, IL, USA). Proportions of the studied variables were expressed as the frequency (%). Continuous data were evaluated for normality using the Shapiro-Wilk test. The variables; age, parity, education level, occupation, history of preeclampsia, history of miscarriage, and BMI were included in the multivariate logistic regression adjusting for possible confounding of the relationship between previous breastfeeding and preeclampsia. All of these variables were included in the regression (i.e. no backward elimination). Some variables such as IPI, blood group, antenatal care, haemoglobin level at delivery, and gender of infant were not included as confounding factors because these variables occurred after the previous breastfeeding. Even if these variables are related to preeclampsia, they are not confounders of the relationship between previous breastfeeding and preeclampsia because they are on the causal pathway. Adjusted odds ratios (ORs) and 95% confidence intervals (CIs) were computed. A *P* value less than 0.05 was considered significant.

## Results

There was no significant difference in the sociodemographic and clinical variables between women who breastfed (*n* = 314) and women who did not breastfeed (*n* = 34) in previous pregnancy (Table 1).

**Table 1:** Comparing the sociodemographic and clinical variables between women who breastfed previous children and women who did not breastfeed previous children

Variables	Women who breastfed (***N*** = 314)	Women who did not breastfeed (***N*** = 34)	Odds Ratio (95% Confidence Interval)
**Age, years**	30.0 (25.0–35.0)	31.0 (25.0–34.0)	1.02 (0.96,1.09)
**Parity**	4.0 (3.0–5.0)	3.0 (2.0–5.0)	1.09 (0.91,1.31)
**Education level**	Numbers (proportion)	Numbers (proportion)	
≥ secondary	182 (58.0)	17 (50.0)	Reference
< secondary	132 (42.0)	17 (50.0)	1.37 (0.67,2.80)
**Occupation**			
Housewife	266 (84.7)	28 (82.4)	Reference
Employee	48 (15.3)	6 (17.6)	1.18 (0.46,3.02)
**History of preeclampsia**			
No	46 (14.6)	8 (23.5)	Reference
Yes	268 (85.4)	26 (76.5)	0.73 (0.38,1.39)
**History of miscarriage**			
No	235 (73.3)	20 (58.8)	Reference
Yes	79 (25.2)	14 (41.2)	1.08 (0.98,1.19)
**Body mass index groups**			
Underweight and normal weight	123 (39.1)	11 (32.3)	Reference
Overweight	126 (40.1)	13 (38.2)	0.92 (0.44,1.91)
Obese	65 (20.7)	10 (29.4)	1.59 (0.69,3.46)

Table 2 shows comparisons of the sociodemographic variables between women with preeclampsia (*n* = 116) and the controls (*n* = 232). Age and parity were significantly higher in women with preeclampsia. More women with preeclampsia were employed and breastfed. There were no significant differences in the educational level, antenatal care attendance, IPI, history of preeclampsia, history of miscarriage, BMI, haemoglobin level, or blood group of the mother or gender of the newborn between the groups. The median (interquartile range) of the duration of breastfeeding in those who breastfed (i.e., excluding those who did not breastfeed) was 4.0 (2.0) months. Among the 116 women with preeclampsia, 26 (22.4%) and 8 (6.8%) had severe and early-onset preeclampsia, respectively.

**Table 2:** Comparing the sociodemographic and clinical variables between women with cases (preeclampsia) and controls

Variables	Cases (preeclampsia, (*n* = 116)	Controls (*n* = 232)	Odds Ratio (95%CI Confidence Interval)	*P*-value
*Variables occurred before the previous breastfeeding*				
**Age, years**	33.0 (28.0–38.0)	30.0 (25.0–33.0)	1.08 (1.05,1.13)	< 0.001
**Parity**	4.0 (3.0–6.0)	3.0 (2–5.0)	1.15 (1.05,1.28)	0.004
**Education level**	Numbers (proportion)	Numbers (proportion)		
≥ secondary	70 (60.3)	129 (55.6)	Reference	0.400
< secondary	46 (39.7)	103 (44.4)	0.82 (0.52,1.29)	
**Occupation**				
Housewife	86 (74.1)	208 (89.7)	Reference	< 0.001
Employee	30 (25.9)	24 (10.3)	3.02 (1.67,5.46)	
**History of preeclampsia**				
No	101 (87.1)	193 (83.2)	Reference	0.347
Yes	15 (12.9)	39 (16.8)	0.73 (0.38,1.39)	
**History of miscarriage**				
No	86 (74.1)	169 (72.8)	Reference	0.898
Yes	30 (25.9)	63 (27.2)	0.93 (0.56,1.55)	
**Body mass index groups**				
Underweight	2 (1.7)	0 (0)	Undefined	
Normal weight	50 (43.1)	82 (35.3)	Reference	
Overweight	40 (34.5)	99 (42.7)	0.77 (0.74,1.40)	0.397
Obese	24 (20.7)	51 (22.0)	0.85 (0.46,1.57)	0.623
*Variables occurred after the previous breastfeeding*				
Interpregnancy interval, months*	22.0 (13.0–36.0)	22.0 (15.0–33.0)	1.01 (0.99,1.02)	0.359
**Antenatal care**				
≥ two visits	106 (91.4)	218 (94.0)	Reference	0.376
< two visits	10 (8.6)	14 (6.0)	1.46 (0.63,3.41)	
**Hemoglobin level, g/dl**	10.6.0 (9.8–11.8)	10.5 (9.9–11.4)	0.96 (0.74,1.24)	0.349
**Blood groups**				
O	53 (45.7)	127 (54.7)	Reference	0.113
Not group O	63 (54.3)	105 (45.3)	1.43 (0.91,2.24)	
**Infant sex**				
Female	58 (50.0)	109 (47.0)	Reference	0.649
Male	58 (50.0)	123 (53.0)	0.88 (0.56,1.38)	

A total of 98 (84.5%) women with preeclampsia and 216 (93.1%) women in the control group had breastfed their previous children. The unadjusted odds ratio (OR) is 3.55; 95% confidence interval (CI) 1.64, 7.70. After adjusting for age, parity, education level, occupation, history of preeclampsia, history of miscarriage, BMI groups the adjusted OR is 3.19; 95% CI 1.49, 6.82 (Table 3).

**Table 3:** Multivariate analysis for association of breastfeeding with preeclampsia (*N* = 116)

		Unadjusted OR	95% CI	***P***-value	Adjusted OR	95% CI	***P***-value
**Breastfed previous child**	Yes	Reference			Reference		
	No	3.55	1.64, 7.70	0.001	3.19	1.49, 6.82	0.006

## Discussion

The main findings of the current study were as follows: compared with women who had breastfed their previous children, women who had not previously breastfed had a higher risk of preeclampsia (Adjusted OR 3.19; 95% CI 1.49, 6.82). A previous study showed that in normal-weight women, breastfeeding was associated with lower blood pressure 1 month after delivery [9]. In a meta-analysis, which included 255,271 women, Rameez et al. reported that breastfeeding for > 2 months was associated with a relative risk reduction of 13% (pooled OR 0.87, 95% CI 0.78, 0.97] for hypertension [20]. Zang et al. reported that women who breastfed for 0–6 months, 6–12 months, and > 12 months had a 13, 17, and 21% lower risk of hypertension, respectively, compared to women who did not breastfeed [21]. Schwarz et al. showed that women who breastfed > 12 months had a lower risk (12% lower risk) of hypertension than women who never breastfed [22]. Likewise, another study showed a lower risk of hypertension in women who breastfed for > 6 months compared with parous women who did not breastfeed [23]. Countouris et al. reported that breastfeeding was not associated with postpartum blood pressure in women with preeclampsia or in those who were normotensive during pregnancy [10].

The exact mechanisms of the association between hypertension and breastfeeding remain unknown. However, it is known that lactation improves the cardiometabolic risk profile [24–26]. Breastfeeding has been reported to be associated with less postpartum weight retention in mothers who had a normal weight before pregnancy [27]. Moreover, a favourable cardiometabolic profile and decreased risk of atherosclerosis have been reported in women with a history of normotensive pregnancies who breastfed for any duration compared with women who did not breastfeed [7]. In a meta-analysis, Nguyen and Ding reported protective effects of breastfeeding on cardiovascular health (metabolic syndrome, inflammatory markers, hypertension and cardiovascular disease) [28]. A previous animal study showed that at 9 months post-delivery, non-lactating mice had a significantly lower cardiac output, ejection fraction, fasting glucose level, and low-density lipoprotein level [26]. In addition, breastfeeding may affect factors that influence systolic blood pressure (arterial stiffness and compliance) [29].

Breastfeeding affects several hormones, such as oxytocin [30], prolactin [31], cortisol [32], oestrogen, and progesterone, which might impact blood pressure. Hormonal pathways (specifically oxytocin) may lower postpartum blood pressure by decreasing inflammatory markers [30]. In the central nervous system, mainly in the vagal nuclei and the locus coeruleus, oxytocin might interact with nitric oxide and atrial natriuretic peptide and lower blood pressure [33]. Ghrelin, a hormone that affects appetite and is associated with breastfeeding, can reduce the risk of metabolic diseases [34, 35]. Moreover, obesity and obesity-associated hypertension may be the result of insulin sensitivity [36].

### Limitations

One of the limitations of the current study was that it only investigated the association between breastfeeding and preeclampsia in parous women. As was expected, it was not possible to include primiparous women because they did not have previous baby or had breastfed. A recent meta-analysis showed that primigravidae were associated with preeclampsia [3]. Another limitation was the report of a history of breastfeeding depended on women’s memories, and thus, bias was expected. However, reliable and valid results have been reported with the recall of breastfeeding [37]. It is obvious that the current study was designed and the sample size was calculated for breastfeeding or not and it was not calculated for the duration of the breastfeeding. Hence it lacks the power for the association between preeclampsia and the duration of breastfeeding.

## Conclusion

Breastfeeding might reduce the risk for preeclampsia. Further larger studies are needed.

## Data Availability

The datasets used and/or analyzed during the current study are available from the corresponding author on reasonable request.

## Abbreviations

AOR: Adjusted odds ratio
BMI: Body mass index
CI: Confidence interval
LR: Likelihood ratio
